# Streptococcus Pneumoniae-Associated Hemolytic Uremic Syndrome in the Era of Pneumococcal Vaccine

**DOI:** 10.3390/pathogens10060727

**Published:** 2021-06-09

**Authors:** Hemant S Agarwal, Samir Q Latifi

**Affiliations:** Department of Pediatric Critical Care, Cleveland Clinic Children’s Hospital, 9500 Euclid Avenue Cleveland, Cleveland, OH 44195, USA; latifis@ccf.org

**Keywords:** *Streptococcus pneumoniae*, hemolytic uremic syndrome, *Streptococcus pneumoniae* induced hemolytic uremic syndrome, pneumococcal vaccine, pneumococcal serotypes, plasmapheresis, eculizumab

## Abstract

*Streptococcus pneumoniae*-associated hemolytic uremic syndrome (Sp-HUS) is a serious complication of invasive pneumococcal disease that is associated with increased mortality in the acute phase and morbidity in the long term. Recently, Sp-HUS definition has undergone revision and cases are categorized as definite, probable, and possible, based on less invasive serological investigations that evaluate Thomsen-Friedenreich crypt antigen (T-antigen) activation. In comparison to the pre-vaccine era, Sp-HUS incidence seems to be decreasing after the introduction of 7-serotype valence and 13-serotype valence pneumococcal vaccines in 2000 and 2010, respectively. However, Sp-HUS cases continue to occur secondary to vaccine failure and emergence of non-vaccine/replacement serotypes. No single hypothesis elucidates the molecular basis for Sp-HUS occurrence, although pneumococcal neuraminidase production and formation of T-antigen antibody complexes on susceptible endothelial and red blood cells continues to remain the most acceptable explanation. Management of Sp-HUS patients remains supportive in nature and better outcomes are being reported secondary to earlier recognition, better diagnostic tools and improved medical care. Recently, the addition of eculizumab therapy in the management of Sp-HUS for control of dysregulated complement activity has demonstrated good outcomes, although randomized clinical trials are awaited. A sustained pneumococcal vaccination program and vigilance for replacement serotypes will be the key for persistent reduction in Sp-HUS cases worldwide.

## 1. Introduction

Hemolytic uremic syndrome (HUS) is an acute medical condition that is characterized by the presence of 3 clinical features: microangiopathic hemolytic anemia, thrombocytopenia, and acute kidney injury. It has a case fatality rate of 12%, and it causes long-term residual kidney damage in 30% of survivors [1]. Infection is the most common cause of HUS. Shiga-like toxin (verocytotoxin) producing bacteria, including enterohaemorrhagic *Escherichia coli* (STEC), mostly *E. coli serotype* 0157:H7 or *Shigella dysenteriae type 1* are responsible for 90% of HUS cases [2]. *Streptococcus pneumoniae* is the most common infection that causes non-diarrheal HUS [3,4,5,6,7]. Five percent of HUS cases occur with invasive infection by *Streptococcus pneumoniae*, also known as *Streptococcus pneumoniae*–HUS (Sp-HUS) [8]. The remaining 5% to 10% of HUS cases occur secondary to medical conditions, drugs, inborn errors of metabolism, or complement pathway dysregulation [8,9].

### 1.1. Classification of HUS

There is no universally accepted classification of HUS because a pattern of clinical disease consistent with HUS occurs in a wide range of clinical scenarios. A classification of HUS has been proposed by the European Pediatric Research Group for HUS at two levels: the first by etiology and the second by clinical features or associations [9]. Commonly, ‘typical’ or ‘post-diarrheal’ or ‘D+ HUS’ are terminology used to describe HUS secondary to verocytotoxin producing *Escherichia coli* (STEC), or *Shigella dysenteriae* [10]. ‘Atypical’ or ‘non-diarrhea-associated’ or ‘D- HUS’ are terminology used to describe any form of HUS that is not due to verocytotoxin producing organisms. In this category, atypical HUS is associated with a wide array of causes that include infections (*Streptococcus pneumoniae*, *human immunodeficiency virus*, and *H1N1 influenza* A), a variety of drugs (*cancer chemotherapy*, *ionizing radiation*, *calcineurin inhibitors*, *sirolimus*, *anti-vascular endothelial growth factor agents*), systemic diseases (systemic lupus erythematous and antiphospholipid antibody syndrome, scleroderma), inborn errors of metabolism (methyl malonic aciduria with homocystinuria, cobalamine defects) or inherited or acquired disorders of complement regulation [9]. Gene mutations of complement factor H (FH), factor I (FI), and membrane co-factor protein (MCP) are associated with HUS [11,12]. It has been proposed that the term “complement dysregulation-associated HUS” be used to describe this latter group [10].

### 1.2. Streptococcus Pneumoniae-HUS (Sp-HUS)

The first description of Sp-HUS in medical literature is in 1971 by Fischer et al. [13] It is a concerning disease process as it is associated with an increased rate of morbidity and mortality in comparison to diarrhea-associated HUS or other forms of invasive pneumococcal disease (IPD) [6,14,15,16]. Studies have reported an annual incidence of Sp-HUS between 0.015‒0.065 cases per 100,000 children [17,18]. This incidence may be erroneous as it is a significantly underdiagnosed disease. A lack of clear Sp-HUS case definition, an absence of a specific laboratory test, misdiagnosis of Sp-HUS as pneumococcal sepsis or disseminated intravascular coagulation (DIC) and unfamiliarity of Sp-HUS are responsible for this underreporting [4,7,19]. The prevalence of Sp-HUS is highest in children less than 2 years of age [4]. Complicated pneumonia is the most common IPD seen in 90% of Sp-HUS cases [20,21,22]. Meningitis is the second most frequent IPD linked to Sp-HUS. However, these patients have a higher mortality rate than other IPD conditions (37% vs. 2%) [4,23]. An increased incidence of Sp-HUS has been reported in the presence of loculated fluid collections, such as parapneumonic or subdural empyema, rather than isolated pneumonia or meningitis in IPD patients [4,13,24,25,26,27]. It is believed that these loculated fluid collections are associated with greater production of pneumococcal neuraminidase and enhanced systemic absorption of extracellular products of *Streptococcus pneumoniae* [18,28,29].

### 1.3. Definition of Sp-HUS

There is lack of consistency in the definition of Sp-HUS. The Canadian Paediatric Surveillance Program classified IPD patients with clinical features of HUS, into definite and possible categories [30]. Definite cases were defined as patients who had thrombotic microangiopathy (TMA) on renal biopsy or autopsy. Possible cases were patients in whom renal biopsy could not be undertaken. In these patients, a Delphi process was used to differentiate Sp-HUS from pneumococcal sepsis with secondary organ failures. Presence of culture-proven *Streptococcus pneumoniae* infection or determination of TMA on renal biopsy are prerequisites for defining Sp-HUS in these schemes. Sp-HUS cases rarely occur in isolated *Streptococcus pneumoniae* bacteremia [31]. They are more commonly seen in IPD patients with pneumonia or meningitis, and blood cultures are positive in only 10–30% of patients with pneumonia [32]. Thrombotic microangiopathy is no longer considered a hallmark of Sp-HUS as it is also seen in patients with DIC [8]. The definition schemes have now expanded to include serological tests, such as peanut lectin agglutination test and direct Coombs test, to define Sp-HUS in the absence of tissue diagnosis [4,8,33,34]. Fluorescein-labeled peanut agglutinin has high affinity for Thomsen-Friedenreich antigen (TF-antigen or T-antigen), and a positive agglutination test confirms the presence of T-antigen on tested cells [25]. Likewise, the direct Coombs test detects the binding of anti-T antibodies to recently exposed T-antigen on the red blood cell membrane. A positive peanut lectin agglutination test or direct Coombs test in an IPD patients suggests T-antigen activation and Sp-HUS [25,34,35,36]. Besides, the direct Coombs test is negative in other categories of HUS or in patients with DIC. Coagulation studies have also been included in the revised definition schemes [7,8]. The modified Sp-HUS definition is shown in Table 1 [7,8].

### 1.4. Streptococcus Pneumoniae Vaccines and Serotypes

*Streptococcus pneumoniae* is a common bacterial pathogen that causes a wide variety of invasive diseases in children and adults throughout the world [37]. *Streptococcus pneumoniae* bacteria have a polysaccharide capsule that acts as a virulence factor for the bacteria. Based on the biochemical structure of their capsular polysaccharide, *Streptococcus pneumoniae* are classified into 97 serotypes within 46 serogroups [38]. As recently as 1995, 90% of clinical episodes of IPD in humans were mainly caused by 23 pneumococcal serotypes [39]. The incidence and serotypes causing Sp-HUS correlate with the trends of IPD.

#### 1.4.1. Pre-Pneumococcal Vaccine Time Period

Between 1950 and 1980, pneumococcal diseases, due to epidemic serogroups (1, 2, 3 and 5), significantly decreased to 2%, whereas the incidence of IPD in children by serotypes 4, 6B, 9V, 14, 18C, 19F, and 23F increased from 59% to 87% [40,41,42,43]. Likewise, the reported incidence of Sp-HUS in children in United Kingdom and Utah, United States were 0.014/100,000, and 0.015/100,000, respectively, in the pre-vaccine era [6,18]. The serotypes 3, 6B, 7, 8, 9V, 14, 19, and 23F were responsible for Sp-HUS in this time period [6,7,14,18,29,44,45,46,47,48].

#### 1.4.2. 7-Serotype Valence Pneumococcal Vaccine Time Period

A pneumococcal conjugate vaccine (PCV7, Prevnar^®^, Wyeth) containing the 7 serotypes (4, 6B, 9V, 14, 18C, 19F, and 23F) most commonly responsible for IPD in children was introduced in the infant immunization schedule in the USA in 2000 [49]. Following the introduction of PVC7, the rates of IPD caused by PCV7 serotypes decreased by 85–95%, and the overall effectiveness of PCV7 against IPD irrespective of the serotype was reported in the range of 50 to 70% [49,50,51,52,53]. However, during subsequent years, *Streptococcus pneumoniae* serotype replacements (3, 7F, 19A, 22F, 33F) resulted in an increase in non-PCV7 type IPD [43,53,54,55,56,57]. Similarly, there were numerous reports of an increase in the number of Sp-HUS cases, in the later years of PCV7, that peaked in 2009–2010 [1,17,18]. A study of public health records in United Kingdom, between 2006 and 2016, revealed an increased incidence of 0.25/100,000 of Sp-HUS in the PCV7 time period [1]. In USA, a study in Utah reported an increase in the percentage of IPD cases, complicated by Sp-HUS, from 0.3%, before the introduction of PCV7, to 5.6% during 2000–2008 [18]. An evaluation of hospital discharges from a USA database revealed that the number of children discharged with Sp-HUS, between 1997 and 2009, doubled [17]. Similar to the etiology of IPD in the PCV7 era, serotype replacements (1, 3, 6A, 7F, and 19A) were responsible for Sp-HUS in this time period [7,18,23,33,58,59]. A USA based study revealed that non PVC7 serogroups (3, 7, and 19) were the most common cause for Sp-HUS in the post PCV7 period [58]. Another USA-based single center study reported that all cases of Sp-HUS after the induction of PCV7 were caused by serotype 19A [7]. A study of 12 Sp-HUS patients in United Kingdom, during this period, reported serotypes 1, 3, 14, and 19A to be responsible for Sp-HUS [33]. Another UK based study of public health records reported that 3, 7F, and 19A were the major serotypes causing Sp-HUS in the PCV7 era [1].

#### 1.4.3. 13-Serotype Valence Pneumococcal Vaccine Time Period

With the emergence of PCV7 serotype replacements, and an increase in the incidence of IPD in the later years of PCV7 era, a 13-valent conjugate vaccine (PCV13, Prevnar-13^®^, Pfizer) containing all the PCV7 serotypes and six additional serotypes (1, 3, 5, 6A, 7F, and 19A) responsible for IPD was introduced in 2010 [60]. Following the introduction of PCV13 vaccination, there has been a reduction in the incidence of IPD by 50–64%, more so with an 85–93% reduction in PCV13/nonPCV7-type IPD [60,61,62]. This IPD reduction was largely driven by declines in IPD caused by serotypes 7F and 19A [57,61,63,64]. The most common serotypes causing IPD in the PCV13 vaccination era were non-PCV13-types (8, 12F, 15B, 15C, 22F, 23B, 24F, 33F, 35B, and 38), and PCV13/nonPCV7-types (3, 7F, 19A) [57,61,62,63,64,65]. Similar data on the impact of PCV13 on Sp-HUS is lacking. A single-center, retrospective review of Sp-HUS cases in Australia, between the time period of 1997 and 2016, reported an increase incidence of Sp-HUS cases from 4%, prior to PCV7 vaccination, to 20% in the PCV7 era and 28% in the PCV13 era [59]. Serotype 19A was responsible for five of the seven reported cases in the PCV13 era in this report, and four of these patients had received the full course of the PCV13 vaccination [59]. However, another study assessing public health records in United Kingdom, between the time period of 2006 and 2016, reported a reduction in the incidence of Sp-HUS to 0.08/100,000 during the PCV13 period [1]. The main serotypes causing Sp-HUS in this study included PVC13 serotypes (19A: 40%, 3: 13%, 7F: 13%) and non-PCV13 serotypes (33F: 27%, 22F: 7%) [1]. A USA-based single center study reported 19A as the sole serotype responsible for Sp-HUS in the PCV13 period [7]. Case reports and case series have reported serotypes 3 and 38 to be responsible for Sp-HUS in the PCV13 era [66,67,68]. Serotype 19A continues to remain an important serotype, causing Sp-HUS despite immunization [1,7,59]. Possible factors for serotype 19A prevalence include high nasal carriage of serotype 19A, penicillin non-susceptibility, capsular switching resulting in the acquisition of a new capsular serotype and enhanced expression of neuraminidase [4,7,69,70,71].

### 1.5. Pathogenesis and Pathology of Sp-HUS

The pathophysiology of Sp-HUS at the cellular and molecular levels remains ill-defined. Multiple theories including the role of *Streptococcus pneumoniae* (neuraminidase, surface protein C), immunological factors (Factor H, complement dysregulation), and host genetic factors (complement gene defects) have been proposed for the pathogenesis of Sp-HUS and it remains unclear if any single factor, or a combination of these factors, contributes to the development of Sp-HUS.

#### 1.5.1. Pneumococcal Neuraminidase Activity

*Streptococcus pneumoniae* bacteria express neuraminidase enzymes that cleave glycosidic linkages of neuraminic acids. Neuraminidase production in *Streptococcus pneumoniae* is encoded by three genes (*nan*A, *nan*B, and *nan*C) that are present in varying frequencies [72]. All strains express *nan*A, whereas *nan*B and *nan*C are expressed in 96% and 51% of *Streptococcus pneumoniae* strains, respectively [72]. It is currently believed that pneumococcal neuraminidase plays a central role in the pathogenesis of Sp-HUS [13]. The amount of neuraminidase production, and the level of neuraminidase activity, supposedly influence the likelihood of development of Sp-HUS [73,74]. A heavy bacterial load leads to increased production of neuraminidase and cause Sp-HUS, as evidenced by clinical association of extensive *Streptococcus pneumoniae* infiltration of the lung, pleural space, or subarachnoid space with the development of Sp-HUS [4,19,21,22,24,75]. Besides, laboratory studies have demonstrated greater pneumococcal neuraminidase production in the lung, rather than in the blood, in mouse models and greater in the bacteria growth in biofilms, than in planktonic forms, that advocate a relationship between empyema and Sp-HUS rather than isolated bacteremia [76,77]. Pneumococcal neuraminidase cleaves terminal *N*-acetyl neuraminic acid (sialic acid) from glycoproteins present on erythrocytes, glomerular endothelial cells, renal epithelial cells, and platelets, and exposes the normally hidden cryptic antigen T-antigen/ TF-antigen [13,28,78,79]. Preformed IgM antibodies react to exposed T-antigen on these cells, leading to the initiation of a cascade of events with the development of Sp-HUS [28]. This hypothesis has been supported by a study that revealed a significant association of T-antigen with Sp-HUS in IPD patients [25]. The study incorporated the peanut (*Arachis hypogaea*) lectin agglutinin assay to test for T-antigen, and the study results revealed the presence of T-antigen exposure in all children with Sp-HUS, in 67% children with IPD associated hemolytic anemia, and in only 43% children with uncomplicated IPD [25]. The study concluded that T-antigen exposure had a 100% sensitivity, and 48% specificity, for Sp-HUS in IPD. Presence of T-antigen exposure for Sp-HUS, using the peanut lectin agglutination assay, has also been confirmed by other studies [80]. However, a recent study of 38 IPD patients revealed that only 66% patients showed T-antigen activation (TA). Of the 25 patients with TA positive, 12 developed Sp-HUS whereas only 1 of 13 TA-negative patients developed Sp-HUS (*p* < 0.01). An association of only 12 of 25 TA positive patients with Sp-HUS suggest that T-antigen exposure, by itself, is inadequate to explain the pathogenesis of Sp-HUS [81]. The interaction of T-antigen and preformed IgM antibody in Sp-HUS is also demonstrated by a positive direct Coombs test that is seen in approximately 90% of Sp-HUS patients [7,36]. Recent studies, however, suggest that a positive Coombs test has only 60% sensitivity for Sp-HUS [19,82]. In addition, anti-T IgM antibody is a cold acting antibody that elicits a stronger in vitro reaction at 4 °C than at 37 °C, and it is debatable whether the exposed T-antigen complexes with host IgM anti-T antibody in vivo, leading to immune-mediated polyagglutination and hemolysis [83,84,85]. Studies evaluating the role of different neuraminidase genes in Sp-HUS have also revealed inconsistent findings of the role of neuraminidase in Sp-HUS [80,86,87]. A study assessing *nan*A in Sp-HUS patients did not demonstrate significant differences in *nan*A activity in vitro, or in genetic content, among IPD patients with and without Sp-HUS [86]. Another study found a significantly higher *nan*C carriage rate in IPD with Sp-HUS (89%) versus without Sp-HUS (41%) that was, however, not confirmed by another group [80,87]. Thus, reports and studies in literature support the role of neuraminidase activity, and T-antigen, activation in the pathogenesis of P-HUS, however, they alone do not seem to sufficiently explain the pathophysiology of Sp-HUS.

#### 1.5.2. Factor H

Factor H is a serum glycoprotein composed of 20 complement control proteins (CCPs) that plays a vital role in the regulation of alternate complement pathway [88]. Four CCPs at the N-terminal end of Factor H act as a cofactor for factor I-mediated proteolytic inactivation of C3b. They compete with factor B for C3b binding and accelerate the breakdown of C3bBb C3/C5 convertase into its components [89,90]. The CCPs at position 19–20, in Factor H, have two C3b binding sites and a sialic acid binding site [91]. As a result, Factor H is able to bind to sialic acid residues on endothelial cells and simultaneously engage the C3b molecule, allowing the 4 CCPs at the N-terminal to prevent further activation of the complement cascade. Thus, Factor H plays a vital role in the protection of the host cells from complement mediated damage. Pneumococcal neuraminidase removes sialic acid from endothelial cell surfaces, leading to decreased factor H binding to these cells. This exposes the endothelial cells to unabated complement-mediated damage. Besides, certain pneumococcal serotypes (2 and 3) express proteins, including Hic and pneumococcal surface protein C, which directly bind to Factor H and inhibit its action [23].

#### 1.5.3. Pneumococcal Surface Protein C

A specific role of pneumococcal surface protein C (PspC) has been proposed in the mechanism of Sp-HUS. Pneumococcal surface protein C binds to plasminogen at the bacterial surface and converts it to plasmin. Plasmin degrades fibrinogen, cleaves C3b, and causes an alteration of complement homeostasis with resultant damage to the endothelial cells. This leads to thrombosis and initiation of Sp-HUS pathology [92]. This hypothesis was supported by a study of two Sp-HUS patients that revealed an increased expression of PspC and the clinical Sp-HUS isolates expressed a specific variant of PspC [92].

#### 1.5.4. Complement Dysregulation

Complement pathway plays a vital role in the development of Sp-HUS. It is not known whether host genetic defects in the complement system or acquired complement dysregulation, secondary to *Streptococcus pneumoniae* infection, or a combination of both lead to Sp-HUS. Decreased levels of C3 and C4 have been reported in numerous Sp-HUS patients, in the acute phase, that reflect activation and consumption of both classical (CP) and alternate (AP) complement pathways [92,93,94,95,96]. A detailed study of the complement profile of five Sp-HUS patients recently revealed that three patients had mutations and/or risk haplotypes in the complement genes [95]. Thus, inherent genetic defects of complement proteins may be responsible for the development of Sp-HUS [95]. As mentioned before, preformed anti-T IgM antibodies bind to structures, exposing T-antigens on different cells, and lead to the activation of the CP. In addition, neuraminidase activity causes a loss of sialic acid from endothelial cell surfaces resulting in decreased factor H binding to these cells and activation of the AP. Thus, *Streptococcus pneumoniae* infection leads to acquired dysregulation of complement activation and consumption terminating in the development of Sp-HUS.

#### 1.5.5. Pathology

HUS patients demonstrate thickened vessel walls at the cellular level [97]. The lumina are filled with platelet thrombi leading to partial or complete luminal obstruction. The vascular endothelial cells are detached, and there is swelling and accumulation of material in the subendothelial space [97].

### 1.6. Clinical Features of Sp-HUS

The clinical features of Sp-HUS including oligo-anuric renal failure, anemia and thrombocytopenia, commonly present 3 to 13 days (average of 7 to 9 days), following the onset of pneumococcal infection [23]. Approximately 25% of Sp-HUS patients develop extra-renal complications including hepatitis, cholecystitis, pancreatitis, hearing deficit, and limb ischemia [21,58]. Hepatocellular involvement manifesting as biliary stasis and jaundice is on the rise in Sp-HUS patients [98,99]. Presence of activated T- antigen on hepatocytes has been postulated to cause hepatic dysfunction in Sp-HUS patients [100]. The clinical features of IPD with Sp-HUS may overlap with some of the symptoms of multi-organ failure seen with pneumococcal sepsis that may delay the recognition of Sp-HUS. The dual manifestations of IPD and Sp-HUS tend to make the clinical course of Sp-HUS more severe with increased morbidity and mortality [23,101]. They are usually quite sick on admission and require a pediatric intensive care unit (PICU) stay for a median duration of 19 days [58,102], and 50% of patients require mechanical ventilation for a median duration of 6.6 days [58]. Approximately two-thirds of Sp-HUS patients undergo invasive procedures, including chest tube placement, video-assisted thoracoscopic surgery, lobectomy/pneumonectomy, or pericardiocentesis for management of their necrotizing pneumonia, pleural effusion and/or empyema [58]. In comparison to other HUS patients, Sp-HUS patients demonstrate a greater degree, and a longer duration, of oligo-anuric renal failure, anemia, and thrombocytopenia that is associated with increased need for renal replacement therapy and blood products administration [7,33,58]. More than half of Sp-HUS patients are supported by renal replacement therapy [7,18,22,58,59,101]. The median length of hospital stays vary from 22 to 32 days [7,58,102]. At discharge, 46% of patients have renal recovery with near-normal serum creatinine concentration, 38% have significantly decreased renal function, and 15–23% are discharged on home dialysis [7,33]. Patients who do not require renal replacement therapy during the acute phase generally have good renal recovery and have normal blood pressure at discharge [7].

### 1.7. Diagnosis of Sp-HUS

The diagnosis of Sp-HUS has undergone changes with the revision of Sp-HUS definition over the period of time [4,8,30,33]. Sp-HUS patients are currently classified into definite, possible, or probable categories based on the feasibility of laboratory studies (Table 1). Sp-HUS commonly occurs in patients with IPD such as pneumonia or meningitis and blood cultures are 10–30% positive in patients with pneumonia [32]. Renal biopsy is not undertaken nowadays, as these patients are unstable, have bleeding risk, and thrombotic microangiopathy is no longer pathognomic for Sp-HUS [8]. Hematological and renal tests are undertaken to confirm the presence of HUS (elevated lactate dehydrogenase levels, diminished haptoglobin levels, presence of free plasma hemoglobin, schistocytosis on peripheral blood smear, thrombocytopenia: platelet count < 150,000/mcL, and presence of acute kidney injury: serum creatinine ≥ 1.5 times appropriate for age; ≥25% decrease in glomerular filtration rate) [4,8,33]. Hematological tests should also evaluate the presence or absence of DIC (prolonged prothrombin time, prolonged partial thromboplastin time, decreased fibrinogen level, and elevated d-dimer level). Invasive *Streptococcus pneumoniae* infection is diagnosed by the presence of pneumococcal growth, antigen detection or positive polymerase chain reaction from blood or physiologically sterile biological fluid. In the absence of invasive infection, probable and possible cases of Sp-HUS are diagnosed by fluorescein-labeled peanut lectin (*Arachis hypogaea*) agglutination test or positive direct Coombs tests that are evidence of T-antigen activation [4,8,33]. Demonstration of T-antigen presence by peanut lectin agglutination test is 100% sensitive. However, the specificity is only 48% for Sp-HUS in IPD and this test is not routinely available [25]. Likewise, initial studies reported positive Coombs test in 90% of Sp-HUS patients whereas more recent studies report positivity in 58% of Sp-HUS patients [7,19,36,82,102]. In addition, there is lack of information of positive Coombs test in IPD patients who do not have Sp-HUS.

### 1.8. Management of Sp-HUS

The management of Sp-HUS is primarily supportive. *Streptococcus pneumoniae* infection should be treated and associated pneumonia or meningitis managed appropriately. Antibiotics including vancomycin and an extended-spectrum cephalosporin are recommended to treat critically ill children with possible or proven IPD [103,104]. Recent hospital-based studies of IPD have revealed good susceptibility rate of *Streptococcus pneumoniae* to vancomycin and third generation cephalosporins and resistance to penicillin [105,106]. Sp-HUS related oligo-anuric acute renal failure, anemia, and thrombocytopenia should be addressed. Renal replacement therapy, either as peritoneal dialysis or hemodiafiltration, must be initiated early in the disease process of Sp-HUS patients with severe kidney injury to manage fluid overload, electrolyte imbalances, and provision of optimum nutrition [7,18,22,58,59,102]. Judicious correction of severe anemia and clinically significant thrombocytopenia and bleeding must be done as unwashed blood products and fresh frozen plasma contain pre-formed antibodies, including ant-TF IgM, that may worsen the Sp-HUS disease process [23,107]. Dextran washing of blood products removes more than 95% of plasma that contains these antibodies [79]. If absolutely necessary, such as Sp-HUS patients undergoing procedures, washed red blood cells or platelets must be administered.

#### 1.8.1. Plasma Exchange

Clinical studies have also focused on treatment of the pathophysiologic process of Sp-HUS disease. An attempt has been made to remove or reduce the levels of anti-T antigen antibodies and neuraminidase in Sp-HUS patients with the use of plasmapheresis/plasma exchange (PE) [33,107,108,109]. In a case series, PE was used to treat Sp-HUS disease in 6 of 43 (16%) patients [33]. Albumin was used as replacement fluid in three patients, and low-titer anti-T antibody fresh frozen plasma in two patients. All six Sp-HUS patients who received PE survived [33]. Likewise, isolated case reports of Sp-HUS patients have reported good outcomes of severe Sp-HUS children after PE administration [107,108,109]. However, there has been a concern for using this modality of treatment, as donor plasma demonstrates the presence of anti-T IgM class antibodies that may interact with T antigen, in Sp-HUS patients, and worsen the pre-existing hemolysis [110]. It is also not well known whether anti-T IgM antibodies are present primarily in the intravascular space that can then be efficiently removed by PE [111]. Currently, the indication for plasmapheresis use is labelled as category III [33,110]. The American Society for Apheresis reports insufficient evidence to establish the efficacy or the risk/benefit ratio of PE [112].

#### 1.8.2. Eculizumab

Eculizumab (Soliris^®^, Alexion Pharmaceuticals, Cheshire, CT, USA) is a recombinant, humanized, monoclonal immunoglobulin G antibody that binds to the complement protein C5 [113]. It inhibits the breakdown of C5 to C5a and C5b resulting in lack of generation of the proinflammatory peptide C5a and the cytotoxic membrane attack complex (MAC) (C5b-9) [113]. Sp-HUS patients demonstrate unregulated complement activity, and there may be a role for the use of eculizumab in these patients, as it blocks the activation of the terminal complement pathway and prevents complement mediated damage. Case series and isolated case reports have reported good outcomes in severe Sp-HUS cases following eculizumab administration [66,96,114,115]. Eculizumab was administered to 3 patients in a case series of seven Sp-HUS patients between 1996 and 2019 [66]. All three patients had low serum C3 levels and did not demonstrate any defects in complement-regulating genes. Eculizumab therapy was stopped in one patient, due to presence of severe infection, and was continued in the other two patients for a period of one to six months. There was rapid recovery of platelet count, and improvement of lactate dehydrogenase, following the first dose of eculizumab in these patients. They came off renal replacement therapy within a month of initiation of eculizumab and had normal to near normal renal function on long term follow-up [66]. Similarly, eculizumab has demonstrated good response in isolated case reports of both pediatric and adult Sp-HUS patients [96,114,115]. Two pediatric Sp-HUS patients, with low C3 level in one patient and low C3 and C4 levels in another patient, with no detectable abnormalities in the complement pathway received eculizumab therapy in the acute phase. There was rapid recovery of their platelet count and neuro-irritability after initiation of eculizumab. Both patients received 4 doses of eculizumab. They demonstrated good renal recovery, renal replacement therapy was stopped within 15–30 days, and they had near normal to normal renal function on long term follow up [96,115]. In another case report of a 53 year old adult Sp-HUS patient, with normal complement factor levels and no defects of complement-regulating genes; eculizumab was initiated after failure of conventional therapy and plasma exchange [114]. There was rapid improvement in his platelet count and mental confusion after the first dose of eculizumab. He came off dialysis by day 14 of therapy. He received eculizumab for 6 months and had normal renal function at discharge and on long term follow up [114]. However, in a case series of seven patients in the time period between 2004 and 2019, two patients with C3 consumption received eculizumab therapy (1 to 3 doses) without any improvement in their clinical course [116]. Based on recent case reports and case series, there may be a role for use of eculizumab in severe cases of S-HUS especially those with reduced complement function. Recommendation for eculizumab use as standard of care for Sp-HUS, however, awaits well-designed clinical trials.

### 1.9. Outcomes of Sp-HUS

The morbidity and mortality associated with Sp-HUS has improved over the period of time. Earlier studies of Sp-HUS patients, between 1977 and 1987, reported that half of Sp-HUS patients died in the acute phase, and in the remaining half who survived, 67% developed chronic renal disease or hypertension [28,117,118]. Advances in the supportive care, and earlier diagnosis, of Sp-HUS have facilitated a dramatic decline in morbidity and mortality of Sp-HUS patients in recent years [4,18,58]. Mortality has been reported in 11–16% of Sp-HUS patients, and it occurs mostly in the acute phase of the illness in patients presenting with meningitis [4,22,33,102]. Between twenty-five and forty-five percent of Sp-HUS patients develop chronic renal sequelae [1,7,18,58]. The severity of the renal injury, and the requirement of renal replacement therapy for more than 20 days in the acute phase, are risk factors for development of chronic renal disease [7]. Approximately 20–28% of patients develop proteinuria, and 19–30% of patients develop hypertension [7,58]. Around ten percent of Sp-HUS patients develop end-stage renal disease and approximately 10% of patients receive renal transplantation [7,58]. Sp-HUS is a single event disease process and no recurrences have been reported.

## 2. Materials and Methods

For this review, a literature search was conducted using PubMed (http://www.ncbi.nlm.nih.gov; accessed on 25 September 2020) and Google Scholar. The key words identified that were relevant for this search included “Streptococcus pneumoniae”, “hemolytic uremic syndrome”, “Streptococcus pneumoniae induced hemolytic uremic syndrome”, “invasive pneumococcal disease”, “pneumococcal conjugate vaccine”, “pneumococcal serotypes” “thrombotic microangiopathy” “T-antigen”, “complement” “eculizumab”, “plasmapheresis” “classification”, and “outcome”. Based on these search terms, the following search string was generated: “hemolytic uremic syndrome” AND (“Streptococcus pneumoniae” OR “invasive pneumococcal disease” OR “pneumococcal conjugate vaccine” OR “pneumococcal serotypes” OR “T-antigen” OR “complement” OR “eculizumab” OR “outcome”). The timeframe was not specified, therefore accessing all records available from the two search platforms were utilized. The titles and abstracts of the search results were then examined for relevance, and appropriate ones were examined in more detail.

## 3. Conclusions

In this review, we conclude that Sp-HUS continues to remain a severe complication of IPD with higher incidence of mortality during the initial presentation and risk of developing chronic renal disease in the long term. More cases of Sp-HUS are being identified nowadays due to better definition schemes and inclusion of less invasive tests such as Coombs test and peanut lectin agglutination test as compared to renal biopsy. The development and incorporation of 7-valence and 13-valence pneumococcal conjugate vaccines in routine childhood immunization schedules in 2000 and 2010, respectively, have resulted in a significant decrease in the incidence of IPD and associated decline in the number of Sp-HUS cases. However, Sp-HUS continue to occur secondary to vaccine failure and emergence of new replacement serotypes. The precise pathophysiology of Sp-HUS at the molecular level continues to unravel although increased production of pneumococcal neuraminidase remains the central hypothesis postulated to cause Sp-HUS disease. Sp-HUS management remains supportive in nature with focus on the eradication of *Streptococcus pneumoniae* infection and management of kidney and hematological injuries. Recent use of eculizumab, for control of dysregulated complement activity in the treatment of Sp-HUS, has been reported in case series and case reports with good outcomes. However, randomized clinical trials of eculizumab therapy for Sp-HUS are awaited.

## 4. For the Future

Sp-HUS will continue to manifest as a severe complication of IPD in young children. There has to be a multi-pronged approach to manage Sp-HUS with inclusion of preventive and newer therapeutic modalities for Sp-HUS. Ensuring a universal vaccination program for children will continue to be an important tool in the reduction in the incidence of IPD and its associated complications, including Sp-HUS [119,120]. A vigilance needs to be maintained for the emergence of resistant and/or newer pneumococcal strains with increased vaccination coverage [121]. The pathophysiology of Sp-HUS is still not well defined. More research will be needed to elucidate the molecular biology of Sp-HUS and develop target specific therapies to improve outcomes.

## Figures and Tables

**Table 1:** *Streptococcus Pneumoniae*-Associated Hemolytic Uremic Syndrome Case Definitions (adapted from references: [7,8]).

Definite Case
- Presence of HUS ^1^ - Presence of invasive *Streptococcus* *pneumoniae* infection ^2^ - Absence of DIC ^3^
Probable case
- Presence of HUS ^1^ - Presence of invasive *Streptococcus* *pneumoniae* infection ^2^ - Presence of DIC ^3^ - Presence of T-antigen activation ^4^
Possible case
- Presence of HUS ^1^ - Presence of pneumonia, meningitis, or other evidence of invasive infection without identification of a specific organism - Presence or absence of DIC ^3^ - Presence of T-antigen activation ^4^

HUS: Hemolytic uremic syndrome; DIC: disseminated intravascular coagulation; T-antigen: Thomsen-Friedenreich antigen. ^1^ HUS: laboratory tests reveal triad of (1) Microangiopathic hemolytic anemia (Increased serum lactate dehydrogenase levels, decreased haptoglobin levels, presence of plasma free hemoglobin, presence of schistocytes on peripheral blood smear); (2) Thrombocytopenia (Platelet count < 150,000/mcL); (3) Acute kidney injury (serum creatinine ≥ 1.5 times appropriate for age: ≥25% decrease in glomerular filtration rate). ^2^ Invasive *Streptococcus pneumoniae* infection: microbiologic tests reveal *Streptococcus pneumoniae* growth or *Streptococcus pneumoniae* antigen detection or positive *Streptococcus pneumoniae* polymerase chain reaction from blood or physiologically sterile biological fluid. ^3^ DIC: laboratory tests reveal prolonged prothrombin time, prolonged partial thromboplastin time, decreased fibrinogen level, elevated d-dimer level at the time of diagnosis. ^4^ T-antigen activation: laboratory tests reveal positive peanut lectin agglutination test or direct Coombs test.

## Data Availability

No new data were created or analyzed in this study. Data sharing is not applicable to this article.
